# *Plasmodium knowlesi* Infection in Traveler Returning to Canada from the Philippines, 2023

**DOI:** 10.3201/eid2910.230809

**Published:** 2023-10

**Authors:** Calvin Ka-Fung Lo, Katherine Plewes, Sakuntla Sharma, Alicia Low, LingHui D. Su, Sara Belga, Ferdinand V. Salazar, Jan Hajek, Muhammad Morshed, Catherine A. Hogan

**Affiliations:** University of British Columbia, Vancouver, British Columbia, Canada (C. Ka-Fung Lo, K. Plewes, S. Belga, J. Hajek, M. Morshed, C.A. Hogan);; Mahidol–Oxford Tropical Medicine Research Unit, Mahidol University, Bangkok, Thailand (K. Plewes);; Centre for Tropical Medicine and Global Health, Nuffield Department of Medicine, University of Oxford, Oxford, UK (K. Plewes);; British Columbia Centre for Disease Control, Vancouver (S. Sharma, A. Low, L.D. Su, M. Morshed, C.A. Hogan);; Research Institute of Tropical Medicine, Muntinlupa, the Philippines (F.V. Salazar)

**Keywords:** *Plasmodium knowlesi*, Canada, the Philippines, Palawan Island, malaria, epidemiology, South-East Asia, parasites

## Abstract

A 55-year-old man sought treatment for an uncomplicated febrile illness after returning to Canada from the Philippines. A suspected diagnosis of *Plasmodium knowlesi* infection was confirmed by PCR, and treatment with atovaquone/proguanil brought successful recovery. We review the evolving epidemiology of *P. knowlesi* malaria in the Philippines, specifically within Palawan Island.

In February 2023, a 55-year-old man sought care at the emergency department of Vancouver General Hospital, Vancouver, British Columbia, Canada, for daily fevers, headache, and abdominal pain 5 days after returning from a 3-week trip to the Philippines. He stayed mostly in Manila but spent 4 days on Palawan Island in the western Philippines 4 days before his return to Canada; he had not taken malaria chemoprophylaxis. Bloodwork was notable for platelet nadir of 48 × 10^9^/L (reference range 150–450 × 10^9^/L), alanine transaminase of 329 U/L (reference range 10–55 U/L), and alkaline phosphatase of 177 U/L (reference range 30–135 U/L). Results of abdominal computed tomography were unremarkable and of a single-target *Plasmodium falciparum* histidine-rich protein 2 rapid diagnostic test were negative. Peripheral blood thin smear demonstrated variable intraerythrocytic parasite morphology, including band-like forms suggestive of *P. malariae* (<0.1% parasitemia) (Figure, panels A–C). Loop-mediated isothermal amplification testing was positive for *Plasmodium* spp. Given absence of criteria for severe malaria, he was discharged with a 3-day course of atovaquone/proguanil (250 mg/100 mg, 4×/d).

**Figure:**
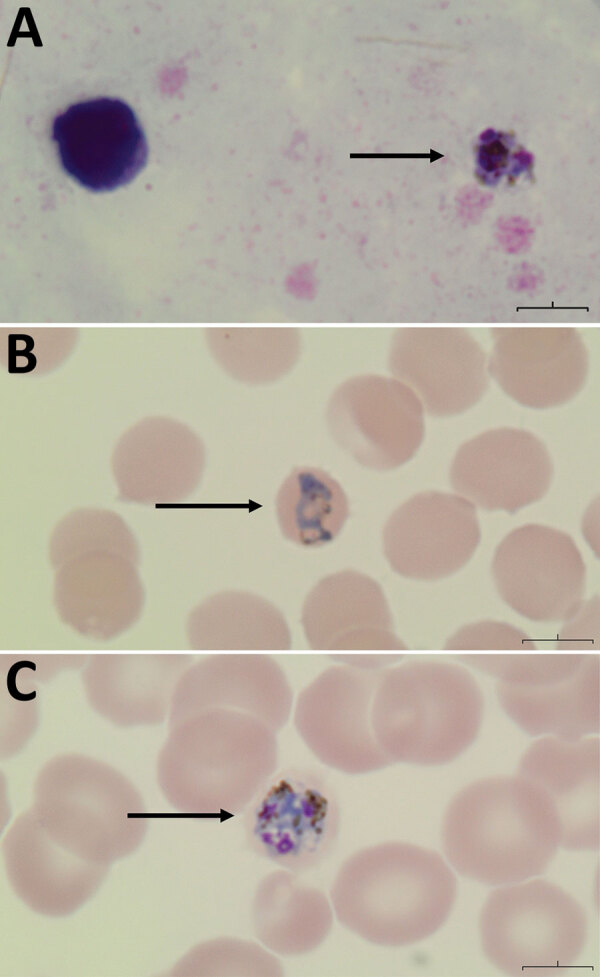
Peripheral thick and thin blood smears of a man in British Columbia, Canada, with suspected *Plasmodium knowlesi* infection after travel to the Phillippines. A) Thick smear showing *P. knowlesi* gametocyte.. B) Thin smear showing band form within a normal-sized, fimbriated erythrocyte with vacuoles present, similar to *P. malariae*. C) Thin smear showing *P. knowlesi* schizont form with presence of greenish-black pigment and lack of rosette formation. Original magnification x100 for all smears.

We suspected a diagnosis of *P. knowlesi*, given the patient’s travel history and blood smear morphology, and subsequently confirmed the infection via species-specific laboratory-developed PCR. Amplicon sequencing on a 118-bp sequence also confirmed *P. knowlesi* identification. The patient was afebrile at follow-up 4 days after drug therapy, with resolution of thrombocytopenia and symptoms.

*P. knowlesi* malaria cases within the Philippines have been concentrated in Cebu Province and, as in this case, Palawan Island (Appendix Figure) (*1*–*4*). Although *P. knowlesi* is primarily a simian malaria infecting nonhuman primate hosts, there has been clear transmission evidence across Southeast Asia since the large 2004 Malaysian outbreak in Sabah (*5*).

Palawan Island contains a diverse landscape of beaches, karst, and mangrove forests, including its well-known Puerto Princesa Subterranean River National Park. The island provides an ideal feeding and breeding ground for ≈500 long-tailed macaques (*Macaca fascicularis*), the only monkey species naturally found in the Philippines and natural host reservoirs for *P. knowlesi* (*6*). Given the close proximity of the island’s diverse habitats to human settlements and recreation areas, constant contact occurs between forest mosquito vectors, macaque hosts, and, potentially, human hosts (*6*). The first 5 confirmed local cases within Palawan were documented in 2005 (*1*); since 2008, only 2 *P. knowlesi* malaria cases have been documented in North America, both in travelers with implicated exposure from Palawan (Table) (*2*,*7*). According to the Philippines Research Institute of Tropical Medicine, >30 *P. knowlesi* cases have been documented, <5 in tourists traveling to Palawan (Philippines Research Institute of Tropical Medicine, pers. comm., email, 2023 Mar 1). However, the true burden is likely underestimated, given the lack of routine molecular testing for species confirmation in the Philippines. Because early ring-form trophozoites of *P. knowlesi* can resemble *P. falciparum* and developing band-like trophozoites can resemble *P. malariae* (*1*), molecular species confirmation is a highlighted need in areas at risk for *P. knowlesi*.

**Table:** Summary of published *Plasmodium knowlesi* cases in North America imported from the Philippines*

Patient age, y	Patient sex	Year	Geographic location presented	Region of Philippines traveled	Diagnosis	Treatment
50	F	2008 (*2*)	New York, NY, USA	Palawan	PCR failed to identify species; confirmed by 1055-bp PCR product sequencing	Atovaquone/proguanil and primaquine
NA	NA	2018 (*7*)	NA	NA	PCR	Atovaquone/proguanil
55	M	2023 (this case)	Vancouver, British Columbia, Canada	Palawan	PCR; confirmed by 118-bp PCR sequencing	Atovaquone/proguanil

*NA, not available.

The 24-hour *P. knowlesi* erythrocytic cycle is shortest among *Plasmodium* spp., which may contribute to rapid development of high parasitemia (*5*), although this case showed ultra-low parasitemia. The World Health Organization (WHO) recommends that uncomplicated *P. knowlesi* malaria infections acquired in *P. vivax* chloroquine-susceptible regions be treated with an oral artemisinin-combination therapy or chloroquine; cases acquired in *P. vivax* chloroquine-resistant regions should be treated with locally available artemisinin-combination therapy (*8*). Despite limited data regarding antimalarial resistance among *P. knowlesi* parasites, this strategy ensures adequate treatment of undiagnosed mixed infections and simplification of uncomplicated malaria treatment. Intravenous artesunate remains first-line treatment for severe *P. knowlesi* malaria. As in this case, atovaquone/proguanil is also considered reasonable empiric treatment.

The Philippines successfully established 0 indigenous cases of malaria across 78 of 81 provinces in 2019; ≈97% of indigenous cases were *P. falciparum* or *P. vivax*. Recent serologic work showed that 1.1% of personsin Palawan tested positive for *P. knowlesi*–specific PkSERA3ag1 antibody (*9*). Current control strategies for human-species malaria (e.g., insecticide-treated nets, indoor spraying) have limited impact on monkey reservoirs and on forest-dwelling *P. knowlesi* vectors, given limited evidence of indoor biting (*9*,*10*). Palawan and Sabah surveillance data identified that most biting by *Anopheles balabacensis* mosquitoes occurred during 6–10 pm, when many rural residents are still outdoors. Although Palawan reports more sporadic cases than does Malaysia, increased encroachment into deforested areas and close proximity (<100 km) to endemic Sabah raises concern of *P. knowlesi* becoming a predominant species in future years. Ongoing investigations into mosquito behavior implicated with cross-species transmission will help inform appropriate control strategies for *P. knowlesi* and other simian species (*9*).

In summary, *P. knowlesi* malaria should be considered in persons with febrile illness who have traveled to the Philippines (especially Cebu Province and Palawan Island). Because of overlapping microscopy features with *P. falciparum* and *P. malariae*, molecular confirmation is required to enable early diagnosis and appropriate treatment. Despite gains in control of *P. falciparum* and *P. vivax* infection in Southeast Asia, the zoonotic nature of *P. knowlesi* and rise in cases highlight the need for tailored prevention and control strategies.

Appendix More information for *Plasmodium knowlesi* infection in traveler returning to Canada from Philippines, 2023.
