# Supplementary material for: Plasmodium knowlesi Infection in Traveler Returning to Canada from the Philippines, 2023
Source: Emerg Infect Dis. 2023 Oct;29(10):2177–9. doi: 10.3201/eid2910.230809 (PMC10521619; doi:10.3201/eid2910.230809)
Supplement: Appendix — More information for Plasmodium knowlesi infection in traveler returning to Canada from Philippines, 2023. [file 23-0809-Techapp-s1.pdf]

Article DOI: http://doi.org/10.3201/eid2910.230809

Plasmodium knowlesi Infection in Traveler Returning to Canada from the Philippines, 2023

Appendix

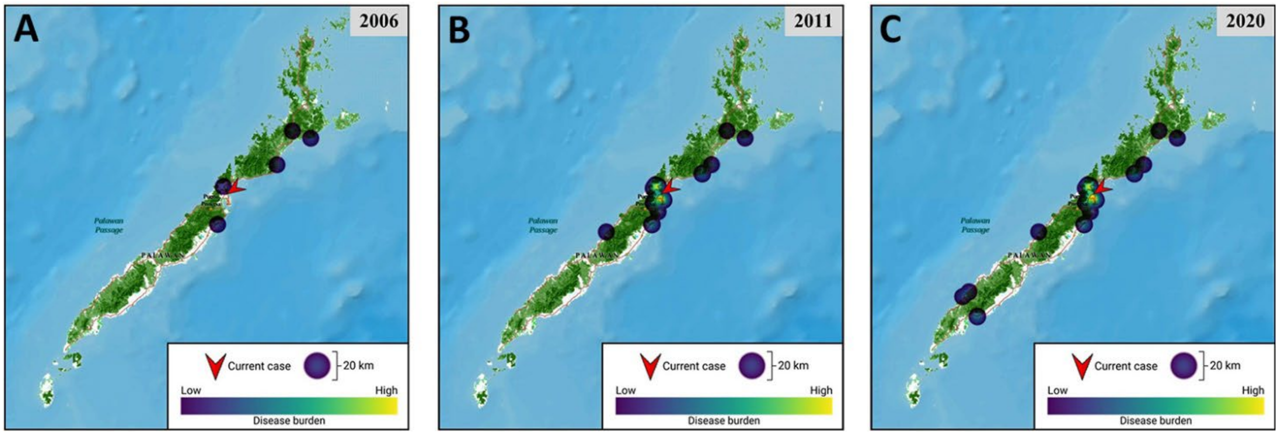

Appendix Figure. Heatmap of cumulative reported human cases of *Plasmodium knowlesi* in Palawan, Philippines. Representation based on unpublished data reported to the Research Institute for Tropical Medicine, the Philippines, at 3 timepoints from 2006, 2011, and 2020 (Appendix Table).

Appendix Table. Reported human cases of *Plasmodium knowlesi* in Palawan, Philippines, at 3 timepoints from 2006, 2011, and 2020

Date of Collection	Year	Residence/ Address (Sitio, Brgy, Municipality)	Latitude	Longitude
6-Jul	2006	Bacungan, PPC	9.909596	118.7012
6-Jul	2006	Balogo, San Miguel, Roxas	10.1159	119.2111
6-Jul	2006	Caibulo, Iraan, Roxas	10.4377	119.358
6-Jul	2006	Inagawan, Tagbarungis, PPC	9.5477	118.6453
6-Jul	2006	Taradungan, Roxas	10.3685	119.5313
16-Nov	2007	San Jose, PPC	9.7944	118.749
22-May	2008	Purok Magalang, Sta. Monica, PPC	9.787061	118.7393
21-May	2008	San Manuel, PPC	9.7793	118.7569
5-Sep	2011	(No data on Sitio) Bacungan, PPC	9.909596	118.7012
9-Feb	2011	(No data on Sitio) Bacungan. PPC	9.909596	118.7012
19-Jul	2011	(No data on Sitio) Inagawan, PPC	9.547699	118.6453
21-Jul	2011	(No data on Sitio) Irawan, PPC	9.810579	118.6946
6-Jun	2011	Berong, Quezon	9.477072	118.2127
9-Jun	2011	Bukang Liwayway, Langogan, PPC	10.03032	119.1235
9-Jun	2011	Iwahig Penal Colony, PPC	9.75028	118.6617
6-Jul	2011	Iwahig Penal Colony, PPC	9.75028	118.6617
16-Feb	2011	Kandis III, Bacungan, PPC	9.891391	118.6571
10-Jun	2011	Langogan, PPC	10.03032	119.1235
30-May	2011	Maranat-3, Bacungan, PPC	9.924164	118.6785
7-Sep	2011	Purok Samplaloc, Irawan, PPC	9.801	118.6971

Date of Collection	Year	Residence/ Address (Sitio, Brgy, Municipality)	Latitude	Longitude
3-Mar	2011	Rubber, Luzviminda, PPC	9.6682	118.7043
2-Jun	2011	San Miguel, PPC	9.7793	118.7569
14-Jul	2014	Candawaga, Bacungan, PPC	8.862	117.4893
13-Aug	2014	Ransang, Rizal	8.90806	117.5502
14-Jul	2014	RJL Marangas, Bataraza+C30	8.6729	117.6292
24-Jul	2014	Ilog-Ilog, Campung Ulay , Rizal	8.9602	117.6143
19-May	2010	Sta. Lourdes	9.8317	118.7222
23-Jul	2014	Tigwayan, Marangas	8.6729	117.6292
